# The Impact of Cytoreductive Surgery and Hyperthermic Intraperitoneal Chemotherapy (CRS-HIPEC) versus Conventional Surgery on Patient-Reported Outcomes: A Comparative Cohort Study between the CAIRO6 Trial and the PROCORE Study

**DOI:** 10.3390/cancers15030788

**Published:** 2023-01-27

**Authors:** Checca Bakkers, Vincent C. J. van de Vlasakker, Koen P. B. Rovers, Robin J. Lurvink, Simon W. Nienhuijs, Jacobus W. A. Burger, Geert-Jan M. Creemers, Cynthia S. Bonhof, Floortje Mols, Ignace H. J. T. de Hingh

**Affiliations:** 1 Department of Surgery, Catharina Cancer Institute, P.O. Box 1350, 5602 ZA Eindhoven, The Netherlands; 2 Department of Medical Oncology, Catharina Cancer Institute, P.O. Box 1350, 5602 ZA Eindhoven, The Netherlands; 3 Center of Research on Psychological and Somatic Disorders, Department of Medical and Clinical Psychology, Tilburg University, P.O. Box 90153, 5000 LE Tilburg, The Netherlands; 4 Department of Research, Netherlands Comprehensive Cancer Organization, P.O. Box 19079, 3501 DB Utrecht, The Netherlands; 5 GROW—School for Oncology and Reproduction, Maastricht University, P.O. Box 616, 6200 MD Maastricht, The Netherlands

**Keywords:** colorectal neoplasms, peritoneal neoplasms, cytoreduction, surgical procedures, CRS-HIPEC, patient-reported outcome measures, quality of life

## Abstract

**Simple Summary:**

Patients treated with cytoreductive surgery and hyperthermic intraperitoneal chemotherapy (CRS-HIPEC) are at risk of significant treatment burden. Multiple studies have reported on patient-reported outcome (PRO) measurements of these patients. However, outcomes are difficult to interpret as no comparison has been made between CRS-HIPEC and conventional surgery. The present study compares several PROs at three different timepoints between patients with colorectal peritoneal metastases treated with CRS-HIPEC and colorectal cancer (CRC) patients treated with conventional surgery. PROs were obtained from two Dutch prospective trials. Despite a more extensive procedure with greater risk of morbidity, CRS-HIPEC in patients with colorectal peritoneal metastases did not have a greater negative impact on PROs than conventional surgery in patients with CRC. Furthermore, systemic therapy did not affect these PROs. These findings may facilitate future patient counseling and shared decision making in clinical practice.

**Abstract:**

Purpose—To compare patient-reported outcomes (PROs) of patients undergoing cytoreductive surgery and hyperthermic intraperitoneal chemotherapy (CRS-HIPEC) for colorectal peritoneal metastases to PROs of colorectal cancer (CRC) patients undergoing conventional surgery. Methods—Data were extracted from the CAIRO6 trial (CRS-HIPEC group) and the PROCORE study (conventional surgery group). Nine predefined PROs (derived from the EORTC QLQ-C30 questionnaire) were compared at baseline, in the early postoperative period and one year postoperatively, with correction for treatment with systemic therapy using linear mixed modeling. Results—In total, 331 patients were included: 71 in the CRS-HIPEC group and 260 in the conventional surgery group. All predefined PROs (fatigue, diarrhea, C30 summary score, Global Health Status, physical, role, emotional, cognitive, and social functioning) did not differ significantly between the groups at all three timepoints, and differential effects over time for all PROs did not differ significantly between the groups. Significant worsening of fatigue, C30 summary score, physical and role functioning (both groups), and cognitive and social functioning (conventional surgery group only) was present in the early postoperative period. All scores returned to baseline at one year postoperatively, except for physical and cognitive functioning in the conventional surgery group. Emotional functioning improved postoperatively in both groups compared to baseline. Conclusion—Despite a more extensive procedure with greater risk of morbidity, CRS-HIPEC in patients with colorectal peritoneal metastases did not have a greater negative impact on PROs than conventional surgery in patients with CRC. Further, systemic therapy did not affect these PROs. These findings may facilitate future patient counseling and shared decision making in clinical practice.

## 1. Introduction

Over the last few decades, treatments for both non-metastatic and metastatic colorectal cancer (CRC) have greatly improved by evolving into multimodality treatments, including surgery, systemic therapy, and/or radiotherapy. This has resulted in prolonged survival and, consequently, with this growing number of CRC survivors, more emphasis on patient-reported outcomes (PROs) is warranted in clinical research [1].

A randomized clinical trial published in 2003 showed that cytoreductive surgery followed by hyperthermic intraperitoneal chemotherapy (CRS-HIPEC) in selected patients resulted in improved survival, as compared to palliative chemotherapy alone [2,3]. Ever since, CRS-HIPEC has been performed with an increasing frequency in patients with colorectal peritoneal metastases and it is recommended in various (inter)national guidelines [4,5].

Although the increasingly frequent application of CRS-HIPEC in this patient group has improved prognosis significantly, it is an invasive treatment with considerable risk of morbidity and mortality [6]. While aiming for macroscopic radical resection of the tumor, CRS-HIPEC usually requires multiple visceral resections, leading to higher risks of morbidity, as compared to conventional surgery for primary CRC [7,8,9,10]. This could result in a significant treatment burden, leading to a decrease in quality of life.

Patients with non-metastatic primary CRC comprise over a million new patients yearly worldwide [11], and PROs are broadly investigated in these patients [12,13]. Likewise, several studies on patients undergoing CRS-HIPEC focusing on PROs have been published [14,15,16,17,18,19,20]. However, no comparative studies are available. Therefore, it remains unknown whether CRS-HIPEC affects PROs more extensively than conventional surgery for primary CRC. In addition, for both patients with primary CRC undergoing conventional surgery and for patients with colorectal peritoneal metastases undergoing CRS-HIPEC, systemic therapy is often part of treatment. This may additionally cause serious side effects, which might consequently affect PROs [21,22,23].

The aims of this study were to compare PROs in patients undergoing CRS-HIPEC for colorectal peritoneal metastases to PROs in patients undergoing conventional surgery for CRC, and to investigate the effect of systemic therapy for these surgical treatments on PROs. Herewith, more insight into the burden of treatment in these patients undergoing extensive treatment for metastatic CRC could be provided.

## 2. Materials and Methods

### 2.1. Study Design and Setting

In this cohort study, PROs of patients undergoing CRS-HIPEC for CPM were compared with PROs of patients undergoing conventional surgery for primary CRC. PROs of patients undergoing CRS-HIPEC for CPM were prospectively collected in the phase 2 part of the CAIRO6 trial [24]. CAIRO6 is an open-label parallel-group trial in all Dutch tertiary centers for the surgical treatment of CPM, with randomization of patients with resectable CPM to perioperative systemic therapy and CRS-HIPEC or upfront CRS-HIPEC alone. PROs of patients undergoing conventional surgery for primary CRC were prospectively collected in the PROCORE study [25]. PROCORE is a population-based, prospective cohort study in four Dutch centers that aims to assess PROs of CRC treatment. The CAIRO6 trial and the PROCORE study were both approved by a central ethics committee (MEC-U, Nieuwegein, Netherlands, numbers NL57644.100.16 (CAIRO6) and NL51119.060.14 (PROCORE)) and the institutional review boards of all participating centers.

### 2.2. Participants

Patients enrolled in CAIRO6 were adults with a WHO performance score of 0–1 and isolated resectable synchronous or metachronous CPM who did not receive systemic therapy ≤6 months prior to enrolment. CAIRO6 patients were included in the present study if they underwent complete CRS-HIPEC. These patients constituted the ‘CRS-HIPEC group’. Patients enrolled in the PROCORE study were adults with newly diagnosed CRC before the start of CRC treatment. To balance patient groups, PROCORE patients were included in the present study if they underwent curative-intent resection for a T3-4N0-2 primary tumor. These patients constituted the ‘conventional surgery group’. Participants in the PROCORE study who underwent CRS-HIPEC for colorectal peritoneal metastases were included as crossovers in the CRS-HIPEC group.

### 2.3. Treatments

All patients in the CRS-HIPEC group underwent cytoreductive surgery (CRS), followed by the perfusion of heated chemotherapy through the peritoneal cavity (HIPEC) to eradicate residual cancer cells. Depending on randomization, patients either underwent CRS-HIPEC alone (control arm of CAIRO6) or CRS-HIPEC with ±3 months of neoadjuvant systemic combination chemotherapy (experimental arm of CAIRO6) (preferentially consisting of capecitabine–oxaliplatin (CAPOX) with bevacizumab, or alternatively consisting of 5-fluorouracil–leucovorin–oxaliplatin (FOLFOX) with bevacizumab or 5-fluorouracil–leucovorin–irinotecan (FOLFIRI) with bevacizumab) and ±3 months of adjuvant systemic combination chemotherapy (consisting of CAPOX, FOLFOX, or 5-fluorouracil).

All patients in the conventional surgery group underwent laparoscopic or open tumor resection (right hemicolectomy, left hemicolectomy, sigmoidectomy, low-anterior resection (LAR), or abdomino-perineal resection (APR)), and (neo)adjuvant systemic therapy (consisting of 5-fluorouracil- or capecitabine-based regimens) if indicated according to the Dutch CRC guideline [26].

### 2.4. PROs

Patients from both groups were asked to complete EORTC QLQ-C30 questionnaire at three time points: at baseline (i.e., before the start of treatment), in the early postoperative period (i.e., ±12 weeks postoperatively in the CRS-HIPEC group, ±5 weeks postoperatively in the conventional surgery group), and one year postoperatively. Scores of all PROs were calculated according to the manuals of EORTC [27,28]. PROs can be divided into function scales (with higher score representing better functioning, e.g., physical functioning) and symptom scales (with higher scores indicating worse symptoms, e.g., fatigue). For the present study, the following PROs were predefined as the most appropriate to assess overall health and treatment tolerability in this setting: C30 summary score, Global Health Scale (GHS), physical functioning, role functioning, emotional functioning, cognitive functioning, social functioning, fatigue, and diarrhea.

### 2.5. Data Collection

Collected patient, tumor, and treatment characteristics included sex, age, American Society of Anesthesiologists (ASA) classification, primary tumor location, and treatment with (neo)adjuvant systemic therapy. For the CRS-HIPEC group, these data were collected using the CAIRO6 trial database. For the conventional surgery group, these data were extracted from the Netherlands Cancer Registry, which registers data from all Dutch patients diagnosed with cancer.

#### Statistical Analyses

All categorical patient and treatment characteristics were compared between the two groups using Chi-square tests. For all patients, mean scores of all PROs at baseline, in the early postoperative period, and one year postoperatively were calculated. Patients who completed questionnaires at least at two time points were included in further analysis. For the comparison of nine predefined PROs between the groups, differential effects over time and scores at each timepoint were compared using linear mixed modeling, with the use of maximum likelihood estimation and an unstructured covariance matrix, with a two-level structure (i.e., repeated timepoints (lower level), patients (higher level)). To adjust for the possible effects of systemic therapy on PROs, the same analyses were performed with correction for treatment with systemic therapy. Within the two groups, longitudinal comparisons between different timepoints were conducted, also using linear mixed modeling. For all PROs being significantly different between the groups, Cohen’s D (CD) effect sizes were calculated to express clinical relevance. CD values ≥ 0.5 were considered clinically relevant. All tests were performed two-sided and pragmatically conducted at *p* < 0.01 to account for multiple testing. All statistical analyses were performed using IBM SPSS Statistics version 25.0 for Windows (IBM Corp, Armonk, NY, USA).

## 3. Results

### 3.1. Patients and Treatments

**Figure 1:**
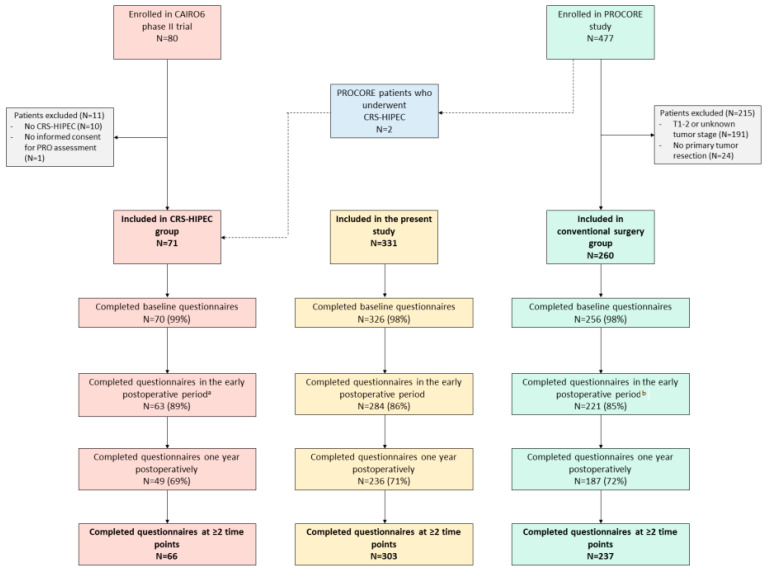
Patient flowchart. CRS-HIPEC cytoreductive surgery and hyperthermic intraperitoneal chemotherapy; a ± 12 weeks postoperatively; b ± 5 weeks postoperatively.

In total, 331 patients were included: 71 in the CRS-HIPEC group and 260 in the conventional surgery group (Figure 1). Patient, tumor, and treatment characteristics are presented in Table 1. Patients in the CRS-HIPEC group were significantly younger than patients in the conventional surgery group (18% vs. 4% ≤50 years, respectively, *p* < 0.001), less frequently had tumors located in the rectum (4% vs. 26% in the conventional surgery group, *p* < 0.001), and more frequently received neoadjuvant systemic therapy (48% vs. 10% in the conventional surgery group, *p* < 0.001). The types of surgery in the conventional surgery group were right hemicolectomy (*n* = 93), left hemicolectomy (*n* = 23), sigmoid resection (*n* = 70), LAR (*n* = 63), APR (*n* = 7), and (sub)total hemicolectomy (*n* = 4).

### 3.2. Questionnaire Completion Rates

In the CRS-HIPEC group, 99% of patients (70/71) completed baseline questionnaires, 89% of patients (63/71) completed questionnaires in the early postoperative period, and 69% of patients (49/71) completed questionnaires one year postoperatively. In the conventional surgery group, 98% of patients (256/260) completed baseline questionnaires, 85% of patients (221/260) completed questionnaires in the early postoperative period, and 72% of patients (187/260) completed questionnaires one year postoperatively.

### 3.3. Comparative Analyses of PROs between Groups and Longitudinal Comparisons within the Groups

**Figure 2:**
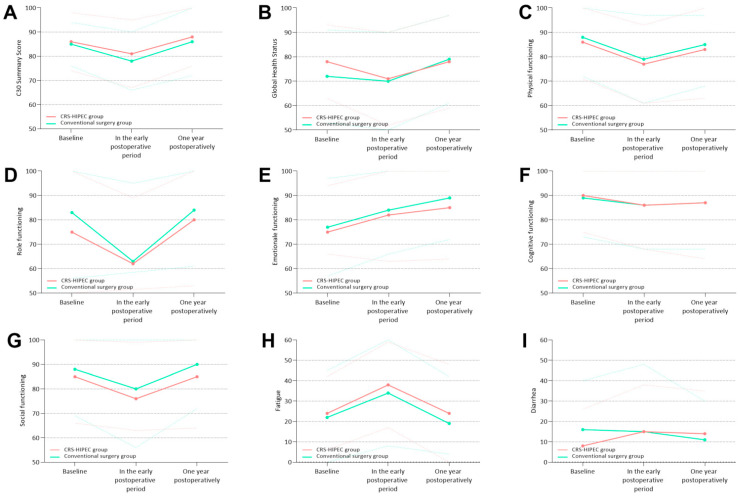
Comparison of predefined patient-reported outcomes between the CRS-HIPEC group and the conventional surgery group. Continuous lines represent mean PRO scores; dotted lines represent standard deviations of PRO scores. (**A**) C30 Summary score; (**B**) Global Health Status; (**C**) physical functioning; (**D**) role functioning; (**E**) emotional functioning; (**F**) cognitive functioning; (**G**) social functioning; (**H**) fatigue; (**I**) diarrhea.

Comparisons and course of all predefined PROs between both groups are presented in Figure 2, with corresponding linear mixed models in Table 2. Longitudinal comparisons within groups are presented in Table 3.

### 3.4. Functional Scales

#### 3.4.1. C30 Summary Score

Differential effects over time (*p* = 0.015) and scores at each timepoint did not differ significantly between the groups, neither before nor after adjustment for systemic therapy (Figure 2A, Table 2). In both groups, the C30 summary score worsened in the early postoperative period (mean difference (MD) −7 (95% CI −11–−4), *p* < 0.001, clinically relevant (CD 0.66), in the CRS-HIPEC group; MD −5 (95% CI −7–−3), *p* < 0.001, non-clinically relevant (CD 0.38) in the conventional surgery group) but returned to baseline values at one year postoperatively (Table 3).

#### 3.4.2. Global Health Status

Differential effects over time (*p* = 0.811) and scores at each timepoint did not differ significantly between the groups, neither before nor after adjustment for systemic therapy (Figure 2B, Table 2). No significant worsening of Global Health Status was present in either of the groups in the early postoperative period; however, a significant improvement at one year postoperatively was present when compared to baseline in the conventional surgery group (MD +7 (95% CI 4–10), *p* < 0.001, non-clinically relevant (CD 0.38), Table 3).

#### 3.4.3. Physical Functioning

Differential effects over time (*p* = 0.033) and scores at each timepoint did not differ significantly between the groups, neither before nor after adjustment for systemic therapy (Figure 2C, Table 2). In both groups, physical functioning worsened in the early postoperative period (MD −9 (95% CI −14–−4), *p* < 0.001, clinically relevant (CD 0.58) in the CRS-HIPEC group; MD −8 (95% CI −11–−7), *p* < 0.001, clinically relevant (CD 0.53) in the conventional surgery group). In the CRS-HIPEC group, physical functioning returned to baseline scores at one year postoperatively. In the conventional surgery group, physical functioning remained worsened at one year postoperatively in comparison to baseline scores (MD −3 (95% CI −6–−1), *p* = 0.003, non-clinically relevant (CD 0.18), Table 3).

#### 3.4.4. Role Functioning

Differential effects over time (*p* = 0.029) and scores at each timepoint did not differ significantly between the groups, neither before nor after adjustment for systemic therapy (Figure 2D, Table 2). In both groups, role functioning worsened in the early postoperative period (MD −12 (95% CI −25–−7), *p* < 0.001, clinically relevant (CD 0.50) in the CRS-HIPEC group; MD −20 (95% CI −24–−16), *p* < 0.001, clinically relevant (CD 0.68) in the conventional surgery group), but returned to baseline values at one year postoperatively (Table 3).

#### 3.4.5. Emotional Functioning

Differential effects over time (*p* = 0.059) and scores at each timepoint did not differ significantly between the groups, neither before nor after adjustment for systemic therapy (Figure 2E, Table 2). In the CRS-HIPEC group, no significant differences in emotional functioning were observed in the early postoperative period, but a significant improvement in emotional functioning at one year postoperatively was observed (MD +13 (95% CI 9–15), *p* = 0.007, clinically relevant (CD 0.50)) as compared to baseline. In the conventional surgery group, emotional functioning improved in the early postoperative period (MD +7 (95% CI 4–9), *p* < 0.001, non-clinically relevant (CD 0.37)) and one year postoperatively (MD +8 (95% CI 2–13), *p* < 0.001, clinically relevant (CD 0.65)) as compared to baseline (Table 3).

#### 3.4.6. Cognitive Functioning

Differential effects over time (*p* = 0.701) and scores at each timepoint did not differ significantly between the groups, neither before nor after adjustment for systemic therapy (Figure 2F, Table 2). In the CRS-HIPEC group, scores on cognitive functioning remained stable at all timepoints. In the conventional surgery group, significant worsening of cognitive functioning was present both in the early postoperative period (MD −4 (95% CI −6–−1), *p* = 0.003, non-clinically relevant (CD 0.18)) and one year postoperatively (MD −3 (95% CI −5–0), *p* < 0.001, non-clinically relevant (CD 0.11)) as compared to baseline (Table 3).

#### 3.4.7. Social Functioning

Differential effects over time (*p* = 0.006) and scores at each timepoint did not differ significantly between the groups, neither before nor after adjustment for systemic therapy (Figure 2G, Table 2). In the CRS-HIPEC group, scores on social functioning remained stable at all timepoints. In the conventional surgery group, significant worsening of social functioning was present in the early postoperative period (MD −8 (95% CI −12–−6), *p* < 0.001, non-clinically relevant (CD 0.37)); however, this returned to baseline at one year postoperatively (Table 3).

### 3.5. Symptom Scales

#### 3.5.1. Fatigue

Differential effects over time (*p* = 0.047) and scores at each timepoint did not differ significantly between the groups, neither before nor after correction for treatment with systemic therapy (Figure 2H, Table 2). In both groups, fatigue worsened in the early postoperative period (MD +14 (95% CI 8–20), *p* < 0.001, clinically relevant (CD 0.72) in the CRS-HIPEC group; MD +12 (95% CI 9–16), *p* < 0.001, non-clinically relevant (CD 0.49) in the conventional surgery group), but returned to baseline scores at one year postoperatively (Table 3).

#### 3.5.2. Diarrhea

Differential effects over time (*p* = 0.976) and scores at each timepoint did not differ significantly between the groups, neither before nor after correction for treatment with systemic therapy (Figure 2I, Table 2). No significant worsening of diarrhea was found in either group, neither in the early postoperative period nor one year postoperatively.

## 4. Discussion

This cohort study compared PROs of patients who underwent CRS-HIPEC for colorectal peritoneal metastases to PROs of patients who underwent conventional surgery for CRC. At all timepoints, PROs did not differ significantly between the groups, neither before nor after correction for possible effects of systemic therapy. Therefore, the results of the present study suggest that CRS-HIPEC in patients with colorectal peritoneal metastases does not affect PROs more extensively than conventional surgery in patients with CRC. Further, systemic therapy did not affect these PROs.

With CRS-HIPEC, the aim is to radically resect all of the visible tumor, including the primary tumor if present. This is typically an extensive surgical procedure, including multiple visceral resections, which is associated with a considerably higher risk of severe postoperative morbidity than conventional surgery alone [8,9,10]. Therefore, this could lead to higher treatment and symptom burden. Previous studies investigating PROs in patients undergoing CRS-HIPEC, indeed, showed a worsening of PROs early after surgery with recovery to baseline levels approximately 6 to 12 months postoperatively, which is comparable with the findings in the present study [14,15,16,17,18,19,20]. Although both studies reporting PROs after CRS-HIPEC and studies reporting PROs after curative colon resection for primary CRC are conducted extensively [12,13], no comparative studies are available. Therefore, the present study provides new insight into the burden of treatment in patients undergoing CRS-HIPEC for colorectal peritoneal metastases and may inform both clinicians and patients about the burden of CRS-HIPEC, thereby facilitating future patient counseling. Despite the more extensive treatment in the CRS-HIPEC group, no significant worsening of PROs was observed in comparison to the conventional surgery group. This indicates that CRS-HIPEC does not have a more substantial negative impact on PROs than conventional surgery only.

As the addition of systemic therapy to the surgical treatment of any type of CRC inevitably prolongs and intensifies treatment and possibly leads to (severe) toxicity, systemic therapy may have an impact on PROs during surgical treatment [29]. Therefore, correction for treatment with systemic therapy was performed through comparative linear mixed modeling. However, no significant effect of systemic therapy on PROs was observed in either group.

At one year postoperatively, all PROs in the CRS-HIPEC group had returned to baseline values, but in the conventional surgery group, a worse physical and cognitive functioning remained, as compared to baseline. Even though other researchers have previously described residual cognitive impairment at one and two years after treatment in CRC patients [30], these effects were not clinically relevant in the present study.

Despite being the first comparative study on PROs in patients undergoing CRS-HIPEC or conventional surgery, this study has some limitations. Firstly, patients in the CRS-HIPEC group were significantly younger than patients in the conventional surgery group, which may have affected PROs. Secondly, the selection procedure of patients for the two study groups was different. In the PROCORE study, all patients undergoing conventional surgery for CRC in four hospitals were asked to participate, while the CAIRO6 trial population comprised patients having to meet much stricter criteria, due to its design and experimental interventions. This additionally resulted in different group sizes, being the third limitation in the present study. However, in order to balance both groups as much as possible, only patients diagnosed with T stage 3–4 were selected from the PROCORE study for the conventional surgery group, as the vast majority of patients with colorectal peritoneal metastases also present with T stage 3–4 CRC. Furthermore, appropriate statistical analyses by means of linear mixed modeling were performed to account for small group sizes. Another limitation is that data on postoperative morbidity and on the presence of any type of ostomy, which might have affected PROs (particularly in the early postoperative period), were not available. Another limitation regarding data is the missing data on specific (neo)adjuvant treatment regimens. Although, in general, no significant effect of systemic therapy on PROs was observed in either group, it is possible that PROs were affected to a greater or lesser extent by the specific systemic therapy regimen (e.g., the toxicity profile of oxaliplatin is more strongly associated with neuropathy than that of irinotecan and, as such, oxaliplatin might have affected the PRO physical functioning more strongly). However, as the PROs are affected by multiple variables and since the percentage of patients in each group that received systemic therapy was comparable, it is expected that the possible effect of the specific systemic therapy regimen was minor.

Lastly, patients in the conventional surgery group completed the questionnaire in the early postoperative period about five weeks postoperatively, as compared to about 12 weeks postoperatively in the CRS-HIPEC group. This could have led to a worsened score from PROs in the conventional surgery group at the early postoperative timepoint as compared to the CRS-HIPEC group due to patients being in an earlier postoperative recovery phase.

## 5. Conclusions

Despite a more extensive procedure with greater risk of morbidity, CRS-HIPEC in patients with colorectal peritoneal metastases did not have a greater negative impact on PROs than conventional surgery in patients with CRC. Systemic therapy in addition to surgical treatment did not significantly affect PROs in either of the groups. These results are valuable for patient counselling and support shared decision making for the treatment of patients with colorectal peritoneal metastases.

## Figures and Tables

**Table 1:** Comparison of patient, tumor, and treatment characteristics between both groups.

	CRS-HIPEC Group N = 71	Conventional Surgery Group N = 260	*p*-Value
**Sex, *n* (%)**			0.176
Male	36 (51)	156 (60)	
Female	35 (49)	104 (40)	
**Age, *n* (%)**			<0.001
≤50	13 (18)	11 (4)	
51–70	40 (56)	135 (52)	
>70	18 (25)	114 (44)	
**ASA, *n* (%)**			0.112
1–2	65 (92)	211 (82)	
3–4	6 (8)	40 (15)	
Unknown	-	9 (3)	
**Primary tumor location, *n* (%)**			<0.001
Right colon	24 (34)	96 (37)	
Left colon	42 (59)	94 (36)	
Rectum	3 (4)	68 (26)	
Unknown	2 (3)	2 (1)	
**Any (neo)adjuvant systemic therapy, *n* (%)**			0.133
No	38 (54)	165 (64)	
Yes	33 (47)	95 (36)	
**Neoadjuvant systemic therapy, *n* (%)**			<0.001
No	37 (52)	235 (90)	
Yes	34 (48)	25 (10)	
**Adjuvant systemic therapy, *n* (%)**			0.553
No	49 (69)	189 (73)	
Yes	22 (31)	71 (27)	

CRS-HIPEC: cytoreductive surgery and hyperthermic intraperitoneal chemotherapy, ASA: American Society of Anesthesiologists classification.

**Table 2:** Linear mixed modeling on comparisons between both groups, with and without correction for systemic therapy.

		Unadjusted		Adjusted for Systemic Therapy	
PRO	Mean Difference ^d^	95% CI	*p*-Value	95% CI	*p*-Value
**C30 summary score ^a^**					
**Comparison of differential effects over time between both groups**	NA	NA	0.015	NA	0.012
**Comparisons between the groups at each measurement ^c^**					
Baseline	−1	−6–2	0.337	−6–2	0.312
Early postoperative period ^d^	−3	−8–−1	0.024	−8–−1	0.021
One year postoperatively	−2	−9–−1	0.020	−9–−1	0.017
**Global Health Status ^a^**					
**Comparison of differential effects over time between both groups**	NA	NA	0.811	NA	0.838
**Comparisons between the groups at each measurement ^c^**					
Baseline	+6	−2–9	0.199	−2–9	0.206
Early postoperative period^d^	+1	−5–6	0.736	−5–6	0.753
One year postoperatively	−1	−10–2	0.232	−10–2	0.222
**Physical functioning ^a^**					
**Comparison of differential effects over time between both groups**	NA	NA	0.033	NA	0.026
**Comparisons between the groups at each measurement ^c^**					
Baseline	−2	−9–1	0.116	−9–1	0.102
Early postoperative period ^d^	−3	−9–1	0.105	−9–1	0.090
One year postoperatively	−2	−11–0	0.069	−11–0	0.057
**Role functioning ^a^**					
**Comparison of differential effects over time between both groups**	NA	NA	0.029	NA	0.029
**Comparisons between the groups at each measurement ^c^**					
Baseline	−9	−16–0	0.057	−16–0	0.059
Early postoperative period ^d^	−1	−11–5	0.496	−11–5	0.501
One year postoperatively	−6	−16–2	0.115	−16–2	0.118
**Emotional functioning ^a^**					
**Comparison of differential effects over time between both groups**	NA		0.059	NA	0.048
**Comparisons between the groups at each measurement ^c^**					
Baseline	−1	−8–3	0.359	−8–3	0.329
Early postoperative period^d^	−2	−9–2	0.229	−9–2	0.205
One year postoperatively	−4	−13–−1	0.017	−14–−2	0.014
**Cognitive functioning ^a^**					
**Comparison of differential effects over time between both groups**	NA	NA	0.701	NA	0.690
**Comparisons between the groups at each measurement ^c^**					
Baseline	−1	−5–5	0.940	−5–5	0.932
Early postoperative period^d^	0	−6–4	0.610	−6–4	0.603
One year postoperatively	0	−7–5	0.721	−7–5	0.711
**Social functioning ^a^**					
**Comparison of differential effects over time between both groups**	NA	NA	0.006	NA	0.006
**Comparisons between the groups at each measurement ^c^**					
Baseline	−3	−12–−0	0.042	−12 –−0	0.044
Early postoperative period^d^	−4	−11–1	0.090	−11–1	0.094
One year postoperatively	−5	−15–−2	0.015	−15–−2	0.016
**Fatigue ^b^**					
**Comparison of differential effects over time between both groups**	NA	NA	0.047	NA	0.048
**Comparisons between the groups at each measurement ^c^**					
Baseline	+2	3–10	0.248	−3–10	0.251
Early postoperative period^d^	+4	−1–12	0.105	−1–12	0.107
One year postoperatively	+5	−1–14	0.096	−1–14	0.099
**Diarrhea ^b^**					
**Comparison of differential effects over time between both groups**	NA	NA	0.976	NA	0.958
**Comparisons between the groups at each measurement ^c^**					
Baseline	−7	−12–1	0.083	−12–1	0.086
Early postoperative period^d^	0	−4–9	0.495	−4–9	0.486
One year postoperatively	+3	−2–12	0.189	−2–12	0.183

PRO patient-reported outcome; CI confidence interval; NA not applicable; ^a^ higher scores represent better functioning; ^b^ higher scores represent worse symptoms; ^c^ calculated as CRS-HIPEC as compared to conventional surgery (conventional surgery as reference); ^d^ +/− 12 weeks postoperatively in CRS-HIPEC group, +/− 5 weeks postoperatively in conventional surgery group.

**Table 3:** Linear mixed modeling on comparisons of scores over time within the groups.

	CRS-HIPEC N = 66				Conventional Surgery N = 237			
PRO	Mean Difference ^c^	95% CI	*p*-Value	Cohen’s D ^e^	Mean Difference ^c^	95% CI	*p*-Value	Cohen’s D ^e^
**C30 summary score ^a^**								
**Comparisons between time points within groups**								
Baseline vs. early postoperative period ^d^	−7	−11–−4	**<0.001**	0.66	−5	−7–−3	**<0.001**	0.38
Early postoperative period ^d^ vs. one year postoperatively	+8	3–10	**0.002**	0.61	+7	5–9	**<0.001**	0.54
Baseline vs. one year postoperatively	+1	−5–−3	0.701	NA	+2	0–4	0.018	NA
**Global Health Status ^a^**								
**Comparisons between time points within groups**								
Baseline vs. early postoperative period ^d^	−7	−10–0	0.066	NA	−2	−5–1	0.119	NA
Early postoperative period ^d^ vs. one year postoperatively	+7	−2–10	0.163	NA	+9	6–12	**<0.001**	0.47
Baseline vs. one year postoperatively	0	−6–5	0.815	NA	+7	4–10	**<0.001**	0.38
**Physical functioning ^a^**								
**Comparisons between time points within groups**								
Baseline vs. early postoperative period ^d^	−9	−14–−4	**<0.001**	0.58	−8	−11–−7	**<0.001**	0.53
Early postoperative period ^d^ vs. one year postoperatively	+6	−1–10	0.146	NA	+5	3–8	**<0.001**	0.34
Baseline vs. one year postoperatively	−3	−10–0	0.074	NA	−3	−6–−1	0.003	0.18
**Role functioning ^a^**								
**Comparisons between time points within groups**								
Baseline vs. early postoperative period ^d^	−12	−25–−7	**<0.001**	0.50	−20	−24–−16	**<0.001**	0.68
Early postoperative period ^d^ vs. one year postoperatively	+18	6–26	**0.002**	0.67	+21	15–25	**<0.001**	0.75
Baseline vs. one year postoperatively	+6	−10–9	0.913	NA	+1	−5–5	0.968	NA
**Emotional functioning ^a^**								
**Comparisons between time points within groups**								
Baseline vs. early postoperative period ^d^	+6	1–11	0.012	0.37	+7	4 –9	**<0.001**	0.37
Early postoperative period ^d^ vs. one year postoperatively	+7	−4–7	0.635	NA	+1	3–8	**<0.001**	0.29
Baseline vs. one year postoperatively	+13	9–15	**0.007**	0.50	+8	2–13	**<0.001**	0.65
**Cognitive functioning ^a^**								
**Comparisons between time points within groups**								
Baseline vs. early postoperative period ^d^	−3	−9–0	0.031	NA	−4	−6–−1	0.003	0.18
Early postoperative period ^d^ vs. one year postoperatively	+1	−3–6	0.525	NA	+1	−1–34	**<0.001**	0.05
Baseline vs. one year postoperatively	−2	−8–2	0.193	NA	−3	−5–0	**<0.001**	0.11
**Social functioning ^a^**								
**Comparisons between time points within groups**								
Baseline vs. early postoperative period ^d^	−9	−15–−1	0.018	NA	−8	−12–−6	**<0.001**	0.37
Early postoperative period ^d^ vs. one year postoperatively	+9	−9–6	0.749	NA	+10	7–14	**<0.001**	0.47
Baseline vs. one year postoperatively	0	−1–15	0.073	NA	+2	−2–5	0.323	NA
**Fatigue ^b^**								
**Comparisons between time points within groups**								
Baseline vs. early postoperative period ^d^	+14	8–20	**<0.001**	0.72	+12	9–16	**<0.001**	0.49
Early postoperative period ^d^ vs. one year postoperatively	−14	−20–7	**<0.001**	0.62	−15	−18–−11	**<0.001**	0.61
Baseline vs. one year postoperatively	0	−6–7	0.908	NA	−1	−6–2	0.279	NA
**Diarrhea ^b^**								
**Comparisons between time points within groups**								
Baseline vs. early postoperative period ^d^	+7	2–14	0.012	NA	0	−4–3	0.781	NA
Early postoperative period ^d^ vs. one year postoperatively	−1	−9–6	0.669	NA	−4	−8–0	0.049	NA
Baseline vs. one year postoperatively	+6	0–14	0.064	NA	−4	−8–−1	0.022	NA

PRO patient-reported outcome; CI confidence interval; NA not applicable; ^a^ higher scores represent better functioning; ^b^ higher scores represent worse symptoms; ^c^ calculated as second mentioned (mean) timepoint minus first mentioned (mean) timepoint for longitudinal comparisons; ^d^ +/− 12 weeks postoperatively in CRS-HIPEC group, +/− 5 weeks postoperatively in conventional surgery group; ^e^ Cohen’s D effect sizes were calculated in case of statistically significant differences between two timepoints.

## Data Availability

Data will be made readily available upon written request sent to the corresponding author.
